# A new cytokine‐based dynamic stratification during induction is highly predictive of survivals in acute myeloid leukemia

**DOI:** 10.1002/cam4.3648

**Published:** 2020-12-25

**Authors:** Pierre Peterlin, Joelle Gaschet, Thierry Guillaume, Alice Garnier, Marion Eveillard, Amandine Le Bourgeois, Michel Cherel, Camille Debord, Yannick Le Bris, Olivier Theisen, Catherine Godon, Béatrice Mahé, Viviane Dubruille, Soraya Wuilleme, Cyrille Touzeau, Thomas Gastinne, Nicolas Blin, Anne Lok, Benoît Tessoulin, Steven Le Gouill, Philippe Moreau, Marie‐C Béné, Patrice Chevallier

**Affiliations:** ^1^ Hematology Clinic CHU Nantes France; ^2^ CRCINA INSERM Université d'Angers Université de Nantes Nantes France; ^3^ Hematology Biology CHU Nantes France; ^4^ Nuclear Medicine Unit ICO Cancer Center Gauducheau Saint Herblain France

**Keywords:** acute myeloid leukemia, FLT3 ligand, IL‐6, prognostic biomarker

## Abstract

The aim of this study was to assess the potential impact of the kinetics of serum levels of seven cytokines during induction in acute myeloid leukemia (AML) patients. Indeed, the role of cytokines, in the pathophysiology and response to therapy of AML patients, remains under investigation. Here, we report on the impact of peripheral levels of two cytokines, the Fms‐like tyrosine kinase 3 ligand (FL) and interleukin‐6 (IL‐6), evaluated during first‐line intensive induction. A new risk stratification can be proposed, which supersedes the ELN 2017 classification to predict survivals in AML patients by examining the kinetic profile of these cytokines during the induction phase. It segregates three groups of, respectively, high‐risk, characterized by a stagnation of low FL levels, intermediate risk, with dynamic increasing FL levels and high IL‐6 at day 22, and favorable risk with increasing FL levels but low IL‐6 at day 22.

## Graphical abstract

Here we report on the impact of the peripheral levels of two cytokines, the Fms‐like tyrosine kinase 3 ligand (FL), and interleukin‐6 (IL‐6), evaluated during first‐line intensive induction. Indeed, a new risk stratification can be proposed, which supersedes the ELN 2017 classification to predict survivals in AML patients by examining their profile during the induction phase.

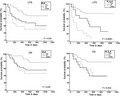

## 1 INTRODUCTION

Several cytokines, such as IL‐1β, IL‐6, or tumor necrosis factor alpha (TNFα), have been proven to contribute to proliferation, blast survival, resistance to treatment, and prognosis in acute myeloid leukemia (AML).^1^, ^2^, ^3^, ^4^ Fms‐like tyrosine kinase 3 ligand (FL), more seldom investigated, is one of the main key regulators of hematopoiesis.^5^ We recently reported on the significance of FL levels in treated AML patients, assessed through the FLAM/FLAL study.^6^ In the latter, three FL kinetic profiles were delineated during induction: (i) sustained increase in FL concentrations between day (D) 1 and D22 (FLI group, *n* = 26, good‐risk); (ii) increase from D1 to D15, then decrease at D22 (FLD group, *n* = 22, intermediate risk); and (iii) stagnation of low levels (<1000 pg/ml, FLL group, *n* = 14, high risk). The impact of FL levels was confirmed independently by a retrospective analysis of the AML17 trial in the UK, showing that a single FL assay at day 26 also predicted survivals in AML patients.^7^ Because serum samples from the FLAM/FLAL study have been frozen stored, we were able to conduct an ancillary study aiming at assessing the potential impact on survivals of the kinetics of other cytokines in the same cohort of AML patients.

## 2 METHODS

The FLAM/FLAL study (ClinicalTrials.gov NCT02693899) included, between May 2016 and January 2018, 62 AML patients at diagnosis (median age 59 years old). All patients received a standard‐of‐care first‐line intensive chemotherapy.^6^ Of eight patients who had FLT3‐ITD mutations, three also received FLT3 inhibitors during induction. Patient characteristics are provided in Table 1. Serum samples were collected and frozen‐stored at D1, D8, D15, & D22 of induction therapy. In these samples, as well as in those of five healthy controls (HC), serum concentrations (pg/ml) of the seven following cytokines were assessed by ELISA (R&D Systems): FL (DY308), TNFα (DY210), stem‐cell factor (SCF) (DY255), IL‐1β (DY201), IL‐6 (DY206), IL‐10 (DY217B), and granulocyte‐monocyte colony‐stimulating factor (GM‐CSF) (DY215). Four patient outcomes were considered, respectively, refractory status after induction, relapse (morphologic, molecular, or immunophenotypic), leukemia‐free (LFS), and overall (OS) survivals.^6^ Factors associated with a *p* value <0.1 in univariate analysis were considered for multivariate analysis. A *p* value <0.05 was considered statistically significant. Analyses were performed using the R and Medcalc (Ostend) software packages.

**TABLE 1:** Patient characteristics

	All patients (*N* = 62)	FLI/FLD with IL‐6 < 15.5 pg/ml D22 (favorable) (*n* = 35)	FLI/FLD with IL‐6 ≥ 15.5 pg/ml D22 (intermediate) (*n* = 13)	FLL (unfavorable) (*n* = 14)	*p* value
Median follow‐up: months (range)	28 (17–37)	28 (17–37)	28.5(17–37)	26.5 (22–33)	0.27
Gender (male)	32	18	2	12	**0.001**
Median age: years (range)	59 (29–71)	60 (29–71)	59 (39–68)	57 (36–66)	0.53
<60 years	33	17	7	9	0.60
ELN 2017 (*n* = 60)					
Favorable (*n*)	23	14	6	3	0.68
Int (*n*)	18	10	4	4	
High (*n*)	19	10	3	6	
WHO AML type					
NOS (*n*)	21	12	3	6	0.54
MDS‐related (*n*)	11	4	3	4	
Rec cyt abn (*n*)	21	15	6	4	
Therapy‐related (*n*)	5	4	1	0	
Bone marrow blasts: median (range)	54 (16–94)	53.5 (16–94)	58.5 (21–94)	51 (25–68)	0.56

Abbreviations: AML, acute myeloid leukemia; D, day; ELN, European Leukemia Net; int, intermediate; MDS, myelodysplastic syndrome; NOS, not otherwise specified; Rec cyt abn, recurrent cytogenetic abnormality; WBC, white blood cell count.

Bold has been used to highlight value that are below 0.05

## 3 RESULTS

Overall, 434 samples were assayed. Median cytokine concentrations for HC and at D1, D8, D15, and D22 for patients are shown in Supporting Information. All median concentrations were 0 pg/ml for HC except for FL and GM‐CSF. In patient samples, median FL concentrations were significantly lower at D1 and higher at D15 and D22 compared to HC. All median IL‐6 concentrations were significantly higher during AML induction compared to HC. Median concentrations of IL‐1β, IL‐10, SCF, and TNFα were at 0 pg/mL all along induction, except for SCF and TNFα at D1. Finally, patient median GM‐CSF concentrations were lower than HC all along induction yet without statistical significance. No particular kinetic profile was disclosed for any of the six cytokines studied other than FL. No significant difference was observed either in terms of median cytokine concentrations at any time when comparing FLI versus FLD versus FLL or FLI versus FLD patients. No difference either was seen between patients with FLT3‐ITD or wild‐type FLT3.

**FIGURE 1:**
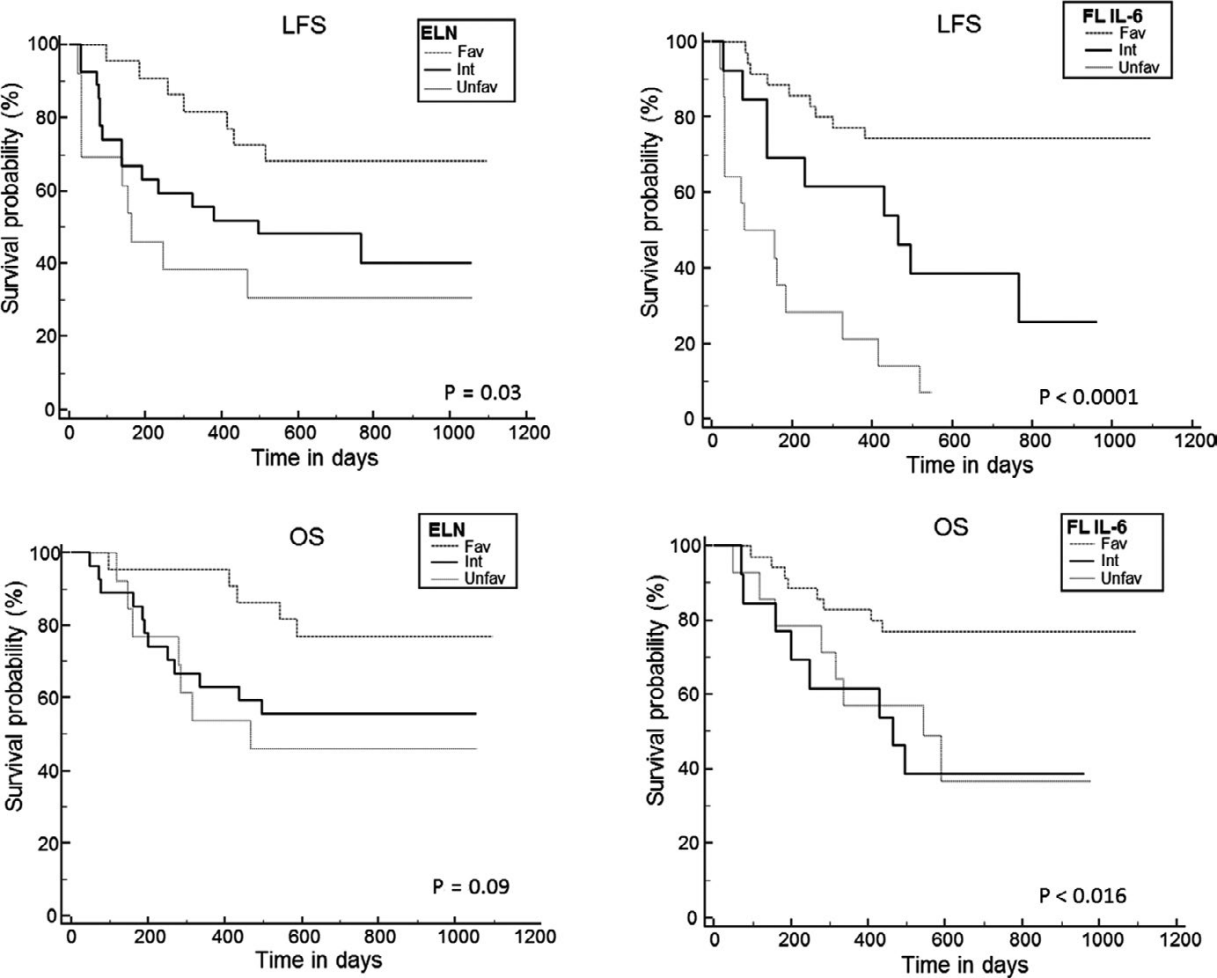
Leukemia‐free survival (LFS), and overall survival (OS). Survivals according to ELN2017 classification (ELN) and to the new cytokine model stratification (FL IL‐6)

With an updated median follow‐up of 28 months (range: 17–37), 2‐year LFS and OS are 51.6% (40–65) and 60.6% (49–74), respectively, for the whole cohort. Of note, OS in the ELN unfavorable‐risk subgroup appears especially good. Usually, unfavorable karyotypes are associated with older age while here, the median age of this subgroup was 61 years old (37–70). Hence, most of these patients (16 of 19) have been allotransplanted, which could explain their favorable outcome. In univariate analysis, FL kinetic profile groups and ELN 2017 classification remained significantly associated with LFS (*p* < 0.001 and *p* = 0.04, respectively) but not OS (*p* = 0.27 and *p* = 0.08) (Table 2; Figure 1). However, with the longer follow‐up, FLI and FLD patients now show similar 2‐year LFS (69.2% vs. 59%, *p* = 0.63) and OS (69.2% vs. 63.6%, *p* = 0.70). FLL patients retain a significantly higher rate of relapse (85.7% vs. FLI 19.2% vs. FLD 32%, *p* = 0.0001). Pooling FLI + FLD patients to compare them with the FLL group disclosed a significantly different LFS (61.1% vs. 7.1%, *p* < 0.001) but not OS (66.6% vs. 36.7% *p* = 0.11).

**TABLE 2:** Univariate analysis, Log rank

*N* = 62	2‐years LFS	*p*	2‐years OS	*p*
Whole cohort	51.6% (40–65)		60.6% (49–74)	
Age: <60 years (*n* = 33) vs. ≥60 years (*n* = 29)	51.5% (36–71) vs. 51.7% (36–73)	0.99	63% (48–82) vs. 58.6% (43–79)	0.48
Gender: M/F	31.3% (18.6–52.2) vs. 73.3 (59.0–88.5)	**0.002**	49.3% (34.5–70.4) vs. 72.8% (58.3–90.9)	0.07
ELN 2017 (*n* = 60)				
Favorable (*n* = 23)	65.5% (48–87)	**0.04**	73.6% (57–94)	0.08
Intermediate (*n* = 18)	55.5% (36–83)		61.1% (42–88)	
High (*n* = 19)	31.5% (16–61)		42.1% (24–71)	
% BM blasts: <median (54%) *n* = 32 vs. median *n* = 30	46.8% (32–67) vs. 56.6% (41–77)	0.39	55.1% (39–76) vs. 66.1% (50–85)	0.26
WBC at diagnosis: <20 10^9^/L *n* = 44 vs. ≥20 10^9^/L *n* = 18	52.2% (39–69) vs. 50% (31–79)	0.93	60.2% (47–77) vs. 61.1% (42–88)	0.90
FL kinetic profile				
FLI (*n* = 26)	69.2% (45–86)	**<0.001**	69.2% (53–89)	0.27
FLD (*n* = 22)	59% (41–83)		63.6% (46–87)	
FLL (*n* = 14)	7.1% (10–47)		36.7% (16–80)	
*p* values of the influence of cytokine levels on survivals				
FL: < vs. ≥ median—Days 1/8/15/22	**0.06/0.03/0.04/0.03**		0.63/0.19/0.44/0.59	
TNF: < vs. ≥ median—Days 1/8/15/22	0.45/0.20/0.42/0.18		0.51/0.20/0.22/0.22	
IL‐6: < vs. ≥ median—Days 1/8/15/22	0.87/0.19/0.92/0.43		0.97/0.32/0.38/0.47	
GMCSF: < vs. ≥ median—Days 1/8/15/22	0.25/**0.04**/0.08/0.90		0.22/0.24/0.08/0.76	
SCF: < vs. ≥ median—Days 1/8/15/22	0.18/0.49/0.99/0.26		0.42/0.94/0.72/0.74	
IL‐10 and IL‐1β	NA (almost all patients with null levels)		NA (almost all patients with null levels)	
New stratification				
FLI/FLD + IL‐6 < 15.5 D22 *n* = 35	74.2% (61–90)	**<0.001**	77.1% (64–92)	**0.01**
FLI/FLD + IL‐6 ≥ 15.5 Day22 *n* = 13	38.4% (19–76)		38.4% (19–76)	
FLL *n* = 14	7.1% (1–47)		36.7% (16–80)	

Abbreviations: BM, bone marrow; ELN, European Leukemia Net; int, intermediate; NA, not applicable; OS, not otherwise specified; WBC, white blood cell count.

Bold has been used to highlight value that are below 0.05

Two‐year LFS and OS were not affected by the concentrations (< or ≥median) of the seven cytokines studied except for LFS, by GM‐CSF at D8, and by FL at D8, D15, and D22. (Table 2). Multivariate analysis showed that no factor was independently associated with OS while LFS was significantly associated with the FL kinetic profile (FLL vs. others, HR: 3.02. 95% CI: 1.77–5.14, *p* < 0.0001) and GM‐CSF levels at D15 (HR: 0.34; 95% CI: 0.15–0.77, *p* = 0.01) but not with the ELN 2017 risk stratification (*p* = 0.055) (Table 3).

**TABLE 3:** Multivariate analysis, Cox proportional regression

Not including the new cytokine risk stratification			
	HR	95% CI	*p* value
OS^a^			
ELN 2017	1.60	0.88–2.89	0.12
GM‐CSF day+15	0.49	0.17–1.40	0.18
LFS^b^			
ELN2017	1.58	0.98–2.52	0.055
FL kinetic profile	3.02	1.77–5.14	**<0.0001**
FL day +1	0.51	0.22–1.15	0.10
GM‐CSF day+15	0.34	0.15–0.77	**0.01**

Including the new cytokine risk stratification			
	HR	95% CI	*p* value
OS^c^			
ELN 2017	1.86	1.03–3.36	0.04
New cytokine risk stratification	3.66	1.36–9.83	**0.009**
LFS^d^			
ELN2017	1.63	0.94–2.83	0.07
New cytokine risk stratification	3.34	1.30–8.54	**0.01**

^a^ Variables not retained in the model: FL kinetic profile, gender.

^b^ Variables not retained in the model: GM‐CSF day +8, FL day+8, +15, and +22, gender.

^c^ Variables not retained in the model: GM‐CSF day +8 and day+15. FL day +1, +8, +15, and +22, gender.

^d^ Variables not retained in the model: GM‐CSF day +15, gender.

Bold has been used to highlight value that are below 0.05

Further analyses were performed to try and better discriminate FLI and FLD patients, who now perform similarly. Between these two subgroups, there was no impact of the concentration (< or >median; data not shown) of the six newly tested cytokines on 2‐year LFS or OS that could keep differentiating them. The population of FLI and FLD (*n* = 48) was thus further assessed as a single group.

Median cytokine concentrations were then compared in this new group of FLI/FLD (that could also be dubbed “non FLL”) patients based on outcome. Relapsed/refractory or deceased patients were compared to alive RC patients. ROC curve analysis showed that the threshold of 15,5 pg/ml of IL‐6 yielded an area under curve (AUC) of 0.68 (*p* = 0.03) with a sensitivity of 50% and specificity of 87%. FLI/FLD patients with low IL‐6 at D22 (< median, 15.5 pg/ml, *n* = 35 vs. *n* = 13 with high levels) had highly significantly better 2 years LFS and OS (74.2% vs. 38.4%, *p* = 0.005 and 77.1% vs. 38.4%, *p* = 0.009, respectively) (Table 2).

A new prognostic risk stratification can thus be proposed as follows: FLI/FLD with D22 IL‐6 < 15.5 pg/ml (favorable), FLI/FLD with D22 IL‐6 ≥ 15.5 pg/ml (intermediate), and FLL (high‐risk). There was no clinical difference between these three groups except for the sex ratio (Table 1). Univariate analysis disclosed strong differences between these three groups in terms of both LFS and OS (Table 2). In multivariate analysis (Table 3), this new cytokine risk stratification was the only factor significantly associated with both OS (HR: 3.66; 95% CI: 1.36–9.83, *p* = 0.009) and LFS (HR: 3.34; 95% CI: 1.30–8.54, *p* = 0.01), while ELN 2017 risk stratification only retained a statistical prognostic significance for OS (HR: 1.86; 95% CI: 1.03–3.36, *p* = 0.03; LFS HR: 1.63; 95%CI: 0.94–2.83, *p* = 0.07).

## 4 DISCUSSION

This study sheds new light on the importance of two cytokines, FL and IL‐6, in the response of AML patients to standard therapy. When FL levels, significantly lower at AML diagnosis than in HC, raise during induction, this is associated with better survival. Moreover, IL‐6 levels, significantly higher all along induction compared to HC, peak by D8 then decrease at D15 and D22. Yet, patients with persisting high IL‐6 levels (above 15.5 pg/ml) at D22, in spite of high FL levels, also display lower survivals. This study confirms our seminal work emphasizing the role of FL levels kinetics during AML induction as a prognostic marker of response to chemotherapy. Some limitations may, however, be noted. First, the measurement of FL levels, although relying on commercially available ELISA tests, is not commonly used. Moreover, no potential confounders such as infections, common in AML patients, have been taken into account here, although they may have interfered with cytokine assessments. Of note, ongoing infection‐related inflammation could be the reason for high D22 IL‐6 levels in the intermediate group.

The deleterious effect of low FL levels is not clearly understood but this situation may sustain blast proliferation instead of normal hematopoietic recovery. Another hypothesis is that persistent leukemia may impair the bone marrow microenvironment to produce FL and/or regenerative cytokines.^8^ Regarding IL‐6, the high levels observed here in AML have been already reported,^1^, ^4^, ^9^ as well as their deleterious impact on outcome.^4^, ^10^ IL‐6 may act via its receptor STAT3, the activity of which is associated with chemoresistance and inferior survival across many malignancies.^11^ That patients with a good outcome manage to lower the IL‐6 response is of particular interest and leads us to propose a new risk stratification for AML patients based on the kinetic profiles of FL and IL6. This stratification appears to be stronger than the ELN 2017 risk stratification which only relies on diagnostic characteristics, while the approach proposed here is more focused on response to therapy. It is likely that stratification criteria will evolve by integrating new parameters such as non‐coding RNA,^12^ and/or by taking into account other parameters such as minimal residual disease.^13^ Of note, peripheral blast cell decrease during induction has already been proposed as an early dynamic prognostic factor.^14^

## 5 CONCLUSION

Cytokine levels assessment during AML induction could emerge as a new valuable monitoring tool. FL and IL‐6 assays, perhaps through the use of fast‐developing microfluidics tests,^15^ could represent a non‐invasive mean to appreciate chemosensitivity in these patients. Of course, these results require to be validated on a larger independent validation cohort of patients. However, it can already be suggested that anti‐IL‐6 or suppletive FL therapy should be tested in combination with standard 3 + 7 chemotherapy in the future.

## CONFLICT OF INTEREST

All authors declare no potential financial conflicts.

## ETHICS APPROVAL AND CONSENT TO PARTICIPATE

The FLAM//FLAL study was registered at ClinicalTrials.gov (NCT02693899). The Ethics committee (Groupe Nantais d'Ethique dans le Domaine de la Santé (GNEDS)) approved the study on the 23th of February 2016 (reference number RC15_0374). All patients include in this study have signed informed consent.

## Supporting information

Supplementary Material

## Data Availability

The datasets used and/or analyzed during the current study are available from the corresponding author on reasonable request.
