# Supplementary material for: A new cytokine‐based dynamic stratification during induction is highly predictive of survivals in acute myeloid leukemia
Source: Cancer Med. 2020 Dec 25;10(2):642–8. doi: 10.1002/cam4.3648 (PMC7877358; doi:10.1002/cam4.3648)
Supplement: Supplementary file 1 — Supplementary Material [file CAM4-10-642-s001.doc]

**SUPPLEMENTAL FILE**

**Median cytokine concentrations (pg/mL, range) during induction.**

A: all cohort n=62.

|  | **Controls**  **N=5** | **Day +1**  **N=62** | **Day +8**  **N=62** | **Day +15**  **N=62** | **Day +22**  **N=62** |
| --- | --- | --- | --- | --- | --- |
| **FlT3-L** | 48.71  (36.17-65.92) | 2 (0-234)  **p=0.01** | 321 (0-7750)  p=0.10 | 2952 (0-14284)  **p=0.004** | 1390 (13-16088)  **p=0.01** |
| **IL-1β** | 0 (0-0) | 0 (0-88.89)  p=0.46 | 0 (0-28.41)  p=0.61 | 0 (0-37.66)  p=0.61 | 0 (0-188.5)  p=0.61 |
| **IL-6** | 0 (0-0) | 4.85 (0-72.5)  **p=0.0008** | 16.28 (0-134.06)  **p=0.0003** | 10.11 (0-181.91)  **p=0.0005** | 7.11 (0-474.26)  **p=0.0006** |
| **IL-10** | 0 (0-0) | 0 (0-436.28)  p=0.30 | 0 (0-92.4)  p=0.68 | 0 (0-132.4)  p=0.68 | 0 (0-296.21)  p=0.51 |
| **GM-CSF** | 10.75 (0-19.5) | 1.63 (0-86.33)  p=0.28 | 1.8 (0-62.67)  p=0.14 | 0.67 (0-44)  p=0.07 | 1.34 (0-28.53)  p=0.11 |
| **TNFα** | 0 (0-0) | 0.53 (0-94.95)  **p=0.03** | 0 (0-46.09)  p=0.15 | 0 (0-43.05)  p=0.20 | 0 (0-19)  p=0.09 |
| **SCF** | 0 (0-11) | 5.91 (0-844.46)  p=0.20 | 0 (0-592.42)  p=0.72 | 0 (0-440.82)  p=0.95 | 0 (0-221.71)  p=0.53 |

p value: comparison of median with controls.

B: FLI+FLD patients n=48

|  | **Day +1**  **N=62** | **Day +8**  **N=62** | **Day +15**  **N=62** | **Day +22**  **N=62** |
| --- | --- | --- | --- | --- |
| **IL-1β** | 0 (0-35.05) | 0 (0-3.04) | 0 (0-1.01) | 0 (0-188.5) |
| **IL-6** | 4.75 (0-65.73) | 14.77 (0-134.06) | 10.11 (0-181.91) | 7.77 (0-474.26) |
| **IL-10** | 0 (0-56) | 0 (0-0) | 0 (0-1.57) | 0 (0-10) |
| **GM-CSF** | 2.06 (0-86.33) | 2.82 (0-62.67) | 0.77 (0-44) | 1.54 (0-28.53) |
| **TNFα** | 0.36 (0-64.57) | 0 (0-46.09) | 0 (0-27.17) | 0 (0-11.3) |
| **SCF** | 6.88 (0-844.46) | 0 (0-592.42) | 0 (0-440.82) | 0.64 (0-221.71) |

**Table 1:** Patient characteristics.

|  | **All patients N=62** | **FLI/FLD with IL-6 <15.5 pg/mL D22 (favorable)**  **(n=35 )** | **FLI/FLD with IL-6 >15.5 pg/mL D22 (intermediate)**  **(n=13 )** | **FLL (unfavorable)**  **(n= 14)** | **P value** |
| --- | --- | --- | --- | --- | --- |
| Median follow-up: months (range) | 28 (17-37) | 28 (17-37) | 28,5(17-37) | 26,5 (22-33) | 0.27 |
| Gender (male) | 32 | 18 | 2 | 12 | **0.001** |
| Median age: years (range)  <60 years | 59 (29-71)  33 | 60 (29-71)  17 | 59 (39-68)  7 | 57 (36-66)  9 | 0.53  0.60 |
| ELN 2017 (n=60)  Favorable n=  Int n=  High n= | 23  18  19 | 14  10  10 | 6  4  3 | 3  4  6 | 0.68 |
| WHO AML type  NOS n=  MDS-related n=  Rec cyt abn n=  Therapy-related n= | 21  11  21  5 | 12  4  15  4 | 3  3  6  1 | 6  4  4  0 | 0.54 |
| Bone marrow blasts: median (range) | 54 (16-94) | 53.5 (16-94) | 58.5 (21-94) | 51 (25-68) | 0.56 |

Abbreviations: ELN: European LeukemiaNet; int: intermediate; NOS: not otherwise specified; MDS: myelodysplastic syndrome; WBC: white blood cell count; Rec cyt abn: recurrent cytogenetic abnormality, AML: acute myeloid leukemia, D: day.
